# Supplementary material for: Complexome profiling of the Chlamydomonas psb28 mutant reveals TEF5 as an early PSII assembly factor
Source: Plant Cell. 2025 Mar 17;37(6):koaf055. doi: 10.1093/plcell/koaf055 (PMC12164590; doi:10.1093/plcell/koaf055)
Supplement: koaf055_Supplementary_Data [file koaf055_supplementary_data.zip › psb28_interactive.zip!/plots/intensity/qualityPlots/peptides/NUOB12_Cre05.g244901.t1.1.html]

### Cre05.g244901.t1.1

Trivial name: NUOB12  
SeqCoverage [median]: 34.50  
Mass: 6.49 kDa  
  
 

| Sequence | MissedCleavage | Mass [Da] | wtIntensity | muIntensity | Score |
| --- | --- | --- | --- | --- | --- |
| HHPLLQFNK | 0 | 1132.61 | 3378.85 | 610.24 | 78.77 |
| PKVVGFQWER | 1 | 1244.67 | 0.00 | 3317.05 | 51.27 |
| VVGFQWER | 0 | 1019.52 | 5056.59 | 5723.83 | 111.01 |
|  |  |  | 8435.4 | 9651.1 |  |
